# The Antibacterial Effects of New *N*-Alkylpyridinium Salts on Planktonic and Biofilm Bacteria

**DOI:** 10.3389/fmicb.2020.573951

**Published:** 2020-10-20

**Authors:** Michaela Hympanova, Saša Terlep, Aneta Markova, Lukáš Prchal, Iztok Dogsa, Lenka Pulkrabkova, Marketa Benkova, Jan Marek, David Stopar

**Affiliations:** ^1^Biomedical Research Centre, University Hospital Hradec Kralove, Hradec Kralove, Czechia; ^2^Department of Epidemiology, Faculty of Military Health Sciences, University of Defence in Brno, Brno, Czechia; ^3^Fotona d.o.o., Ljubljana, Slovenia; ^4^Department of Toxicology and Military Pharmacy, Faculty of Military Health Sciences, University of Defence in Brno, Brno, Czechia; ^5^Department of Microbiology, Biotechnical Faculty, University of Ljubljana, Ljubljana, Slovenia

**Keywords:** antimicrobial activity, quaternary ammonium salts, bacteria, biofilm, photoacoustic irrigation

## Abstract

An increasing microbial resistance to known antibiotics raises a demand for new antimicrobials. In this study the antimicrobial properties of a series of new *N*-Alkylpyridinium quaternary ammonium compounds (QACs) with varying alkyl chain lengths were evaluated for several nosocomial pathogens. The chemical identities of the new QACs were determined by NMR, LC-MS, and HRMS. All the planktonic bacteria tested were susceptible to the new QACs as evaluated by MIC and MBC assays. The antimicrobial effect was most pronounced against *Staphylococcus aureus* clinical isolates. Live/dead staining CLSM was used to test the effectiveness of the QACs in biofilms. The effectiveness was up to 10-fold lower than in the plankton. When QACs were used as irrigants in Er:YAG – SSP photoacoustic steaming, their effectiveness significantly increased. The combined use of irrigants and photoacoustic streaming increased biofilm removal from the surface and increased the killing rate of the cells remaining on the surface. This may allow for a shorter chemical exposure time and lower dosage of QACs used in applications. The results demonstrate that the new QACs have potential to be applied as antibacterial compounds effective against planktonic and biofilm bacteria as well as irrigants in removal of difficult-to-reach biofilms.

## Introduction

Infectious diseases caused by pathogenic bacteria are a threat to public health all over the world (Courvalin, 2016; Watkins, 2018). A high prevalence of resistance against known antibacterial agents aggravates the situation. For instance, many nosocomial pathogens such as *Pseudomonas aeruginosa*, *Staphylococcus aureus*, *Escherichia coli, Klebsiella pneumoniae, and Enterococcus faecalis* are resistant to known antimicrobials and are difficult to treat (Khan et al., 2017). There is an urgent need to develop new antibacterial agents that will replace those to which bacteria have developed resistance. In particular the new antibacterial agents should have good activity against biofilm bacteria which are typically more resistant and are more difficult to remove from surfaces (Ceri et al., 1999; Olson et al., 2002).

Quaternary ammonium compounds (QACs), are widely used in medicine. They have proven antimicrobial properties, have low toxicity, and are minimally irritating (Gerba, 2015). They are most effective against Gram positive bacteria, but are also effective against Gram negative bacteria, viruses and biofilms (Jennings et al., 2014; Gerba, 2015). QACs can be used in antibacterial formulations or incorporated into medical products, such as composite materials or acrylic resins, or adhesive systems and endodontic dental materials, where their antimicrobial activity against *Streptococcus mutans*, *E. faecalis* or other bacteria decreases the occurrence of secondary caries infection (Zhang et al., 2018).

In difficult-to-reach biofilm infections chemical treatments are sometimes combined with physical or mechanical co-treatment methods to increase their effectiveness. For example, in a dental root canal procedure chemical antibacterial treatment (i.e., sodium hypochlorite) is combined with root irrigation. However, sodium hypochlorite is toxic and is not suitable for certain medical applications (i.e., in dental implant related peri-implant mucositis and peri-implantitis). The traditional method of irrigation using a syringe with a needle often fails because of the limited irrigant flow and its ability to reach distant areas (Boutsioukis et al., 2010). Various new irrigant activation techniques have been suggested to improve biofilm removal (Kurzmann et al., 2019). The non-contact Er:YAG photoacoustic streaming with Super Short Pulses (SSP, 50 μs) has been a very successful method of removing biofilms from the dental root system for many years (Olivi et al., 2014; Akcay et al., 2017; Lukač and Jezeršek, 2018; Kurzmann et al., 2019). Er:YAG photoacoustic irrigation causes biofilm mechanical debridement by turbulent movement of fluid irrigant, and at the same time the chemical action of the irrigant itself significantly improves biofilm removal. The problem with irrigation techniques is that many of currently used irrigants in dentistry are toxic in high concentrations (Clarkson and Moule, 1998; Zehnder, 2006). It would therefore be beneficial if new antibacterial irrigants (e.g., QACs) with lower toxicity were available, especially for irrigation in presence of vital tissue. The use of QACs for photoinduced irrigation treatment of biofilms has not been tested yet.

In this study new QACs were evaluated for their efficiency as irrigant and antibacterial compounds against plankton and biofilm. Antibacterial effects were tested on clinical isolates of nosocomial pathogens including *S. aureus, Staphylococcus epidermidis, E. faecalis, E. coli*, and *K. pneumoniae*. For laboratory testing and irrigation experiments non-pathogenic strain of *E. faecalis* was used. The antibacterial effect was probed either by chemical treatment or a combination of chemical and Er:YAG irrigation methods. The chemical identity of the new QACs was determined by NMR, LC-MS, HRMS. The antibacterial activity of QACs was measured with MIC and MBC assays. The results suggest that new QACs have a good potential as antibacterial compounds effective against planktonic and biofilm bacteria, and also as irrigants in laser-assisted removal of biofilms.

## Materials and Methods

### Chemistry

#### General Information

The preparation of 1-Alkylpyridinium derivatives (**A_12_, A_14_, A_16_**) and 3-alkyl-1-(2-hydroxyethyl)imidazolium (**C_12_, C_14_, C_16_**) derivatives with appropriate alkyl chain length (−C_12_H_25_; −C_14_H_29_; -C_16_H_33_) has been described previously (Marek et al., 2010; Soukup et al., 2020). The series of 1-alkyl-3-chloropyridinium derivatives (**B_12_, B_14_, B_16_**) was prepared for the first time in this study by refluxing 3-chloropyridine (18 mM) with the appropriate alkyl bromide (45 mM) in acetonitrile (50 ml) for 96 h. The acetonitrile was evaporated and the crude product purified by recrystallization several times from diethyl ether. Alkyl bromides and 3-chloropyridine were purchased from Sigma-Aldrich (Prague, Czechia) and used without further purification. Acetonitrile was purchased from VWR (Prague, Czechia). The reaction progress was monitored by thin layer chromatography (TLC) on aluminum sheets with silica gel 60 F_254_ purchased from Merck (Prague, Czechia) in a mobile phase of methanol: ethyl acetate: ammonia solution = 3: 1: 0.1. Detection was carried out with ultraviolet light (254 nm) or with KMnO_4_ solution (1% aq. sol.). ^1^H NMR and ^13^C NMR spectra were recorded in CDCl_3_ at ambient temperature on a Varian S500 spectrometer (499.87 MHz for ^1^H and 125.71 MHz for ^13^C). Chemical shifts, δ, are given in parts per million (ppm), and spin multiplicities are given as s (singlet), d (doublet), dd (doublet of doublets), dt (doublets of triplets), t (triplet) or m (multiplet). Coupling constants, *J*, are expressed in hertz (Hz). For ^1^H, δ is relative to CDCl_3_ (δ = 7.26) and for ^13^C relative to CDCl_3_ (δ = 77.00). Melting points were determined by melting point apparatus – Stuart SMP30 (Eaton, United Kingdom) – and were uncorrected.

#### LC-MS Analysis

High performance liquid chromatography (HPLC) coupled with mass spectrometry (MS) detection was performed to determine the identity and purity of the prepared compounds. The system used in this study was a Dionex Ultimate 3000 UHPLC: RS Pump, RS Column Compartment, RS Autosampler, Diode Array Detector, Chromeleon (version 7.2.9 build 11323) software (Thermo Fisher Scientific, Germering, Germany) with Q Exactive Plus Orbitrap mass spectrometer with Thermo Xcalibur (version 3.1.66.10.) software (Thermo Fisher Scientific, Bremen, Germany). Detection was performed by mass spectrometry in positive mode. The settings for the heated electrospray source were: spray voltage 3.5 kV; capillary temperature: 300°C; sheath gas: 55 arbitrary units; auxiliary gas: 15 arbitrary units; spare gas: 3 arbitrary units; probe heater temperature: 250°C; max spray current: 100 μA; S-lens RF Level: 50. High resolution mass spectra (HRMS) and sample purities were obtained by HPLC-MS gradient method. A C18 column was used (Phenomenex Kinetex EVO C18, 3 × 150 mm, 2.6 μm, Phenomenex, Japan). Mobile phase A was ultrapure water of ASTM I type (resistivity 18.2 MΩ.cm at 25°C) prepared by Barnstead Smart2Pure 3 UV/UF apparatus (Thermo Fisher Scientific, Bremen, Germany) with 0.1% (v/v) formic acid; mobile phase B was acetonitrile (MS grade, Honeywell Sigma-Aldrich, Germany) with 0.1% (v/v) of formic acid. The flow was constant at 0.4 mL/min. The method began with 1 min of isocratic flow of 5% B, followed by gradient flow of B rising to 100% B in 3 min, followed by constant flow of 100% B for 1 min. The composition then went back to 5% B and equilibrated for 5 min. Total run time was 10 min. The samples were dissolved in methanol (LC-MS grade, Fluka Sigma-Aldrich, Steinheim, Germany) at a concentration of 1 mg/mL and sample injection was 1 μL. Purity was determined by UV at 254 nm. HRMS was determined by total ion current spectra from the mass spectrometer.

#### NMR and HRMS Data

##### 1-dodecyl-3-chloropyridinium bromide (**B_12_**)

^1^H NMR (500 MHz, methanol-d_4_) δ 9.37 (t, *J* = 1.8 Hz, 1H, ArH), 9.06 (dt, *J* = 6.0, 1.2 Hz, 1H, ArH), 8.73–8.67 (m, 1H, ArH), 8.14 (dd, *J* = 8.5, 6.1 Hz, 1H, ArH), 4.71–4.64 (m, 2H, CH_2_), 2.10–2.01 (m, 2H, CH_2_), 1.48–1.24 (m, 18H, 9 × CH_2_), 0.90 (t, *J* = 7.0 Hz, 3H, CH_3_).

^13^C NMR (126 MHz, CD_3_OD) δ 146.67, 145.53, 144.72, 137.01, 130.04, 63.61, 33.05, 32.42, 30.71, 30.61, 30.47, 30.44, 30.09, 27.16, 23.71, 14.43.

ESI-MS: m/z 282.20 [M^+^] (calc. for [C_17_H_29_ClN^+^] 282.20).

##### 1-tetradecyl-3-chloropyridinium bromide (**B_14_**)

^1^H NMR (500 MHz, chloroform-d) δ 9.70–9.65 (m, 2H, ArH), 8.48 (dd, *J* = 7.8, 1.8 Hz, 1H, ArH), 8.25 (dd, *J* = 8.5, 6.0 Hz, 1H, ArH), 5.10 (t, *J* = 7.5 Hz, 2H, CH_2_), 2.09–1.99 (m, 2H, CH_2_), 1.45–1.14 (m, 22H, 11 × CH_2_), 0.86 (t, *J* = 6.8 Hz, 3H, CH_3_).

^13^C NMR (126 MHz, CDCl_3_) δ 144.97, 144.12, 143.75, 135.65, 129.16, 62.35, 31.98, 31.82, 29.58, 29.54, 29.51, 29.44, 29.27, 29.25, 29.01, 25.97, 22.59, 14.03.

ESI-MS: m/z 310.23[M^+^] (calc. for [C_19_H_33_ClN^+^] 310.23).

##### 1-hexadecyl-3-chloropyridinium bromide (**B_16_**)

^1^H NMR (500 MHz, chloroform-d) δ 9.68 (d, *J* = 6.0 Hz, 1H, ArH), 9.63 (t, *J* = 1.7 Hz, 1H, ArH), 8.48 (dd, *J* = 8.4, 2.0 Hz, 1H, ArH), 8.24 (dd, *J* = 8.5, 6.0 Hz, 1H, ArH), 5.11 (t, *J* = 7.5 Hz, 2H, CH_2_), 2.09–1.99 (m, 2H, CH_2_), 1.46–1.11 (m, 26H, 13 × CH_2_), 0.86 (t, *J* = 6.9 Hz, 3H, CH_3_).

^13^C NMR (126 MHz, CDCl_3_) δ 144.98, 144.16, 143.70, 135.67, 129.15, 62.41, 31.98, 31.85, 29.63, 29.58, 29.54, 29.46, 29.29, 29.03, 25.99, 22.61, 14.05.

ESI-MS: m/z 338.26 [M^+^] (calc. for [C_21_H_37_ClN^+^] 338.26).

### Bacterial Strains

The bacterial strains used in this study are listed in Table 1. All the strains, apart from *E. faecalis*, were stored at the Department of Epidemiology, Faculty of Military Health Sciences, University of Defence in Brno (Czechia) using ITEST CRYOBANK B cryotubes (ITEST plus s.r.o., Hradec Kralove, Czechia) in a freeze box at −70°C. Before MIC/MBC testing by broth microdilution method, all strains were cultivated on Mueller-Hinton agar (HiMedia, Cadersky-Envitek, Prague, Czechia). *Enterococcus faecalis DSM 16431* (kindly donated by SymbioGruppe GmbH & Co KG SymbioPharm GmbH) was stored in cryovials at the Department of Microbiology, Biotechnical Faculty, University of Ljubljana (Slovenia) and was grown on Tryptic Soy Agar (Biolife, Italiana S.r.l., Milan, Italy).

**TABLE 1:** Bacterial strains used in this study.

	***Strain***	**Abb.^a^**	**Source**
**G+**	*Staphylococcus aureus* C1947	STAU	Clinical isolate^b^
	methicillin-resistant *S. aureus* C1926	MRSA	Clinical isolate^b^
	*Staphylococcus epidermidis* C1936	STEP	Clinical isolate^b^
	*Enterococcus faecalis DSM 16431*	EFAE	SymbioGruppe GmbH & Co KG SymbioPharm GmbH)
**G**−	*Escherichia coli* A1235	ESCO	Clinical isolate^b^
	*Klebsiella pneumoniae* C1950	KLPN−	Clinical isolate^b^
	extended-spectrum β-lactamase-producing *K. pneumoniae* C1934	KLPN+	Clinical isolate^b^

*^*a*^abbreviations for Figures and Tables in this publication. ^*b*^linical isolates of patients from the University Hospital Hradec Kralove.*

### Planktonic Bacteria Susceptibility Assay

The antibacterial susceptibility of planktonic bacteria was determined by the broth microdilution method according to the standard M07-A1 (CLSI, 2018) and optimized as described previously (Marek et al., 2015; Dolezal et al., 2016). All antibacterial compounds were dissolved in dimethyl sulfoxide (DMSO p.a., Sigma-Aldrich, Prague, Czechia). The wells of the 96-well microtiter plates contained 200 μL of Mueller-Hinton broth (MHB, HiMedia, Cadersky-Envitek, Prague, Czechia) with two-fold serial dilutions of the QACs (500–0.49 μmol/L) and were inoculated with 10 μL of exponentially grown bacterial suspension adjusted densitometrically to match 0.5 McFarland scale. The final concentrations of DMSO in broth did not exceed 1%. The MIC values, defined as inhibition of bacterial growth, were determined visually after 24 and 48 h of incubation at 35°C ± 1°C. The MBCs were determined for all prepared compounds as the concentrations that provided ≥99.9% decrease in the bacterial number after subculture of 10 μL aliquots from each microtiter well in a corresponding new microtiter plate where each well contained 200 μL of fresh MHB. The MBC was determined as the lowest concentration which corresponded to a well without visible bacterial growth after a further 24 h of incubation at 35 ± 1°C. To ensure that no bacteria had survived, the content of the well was inoculated on an agar plate to confirm the absence of bacterial growth.

To evaluate the effect of bacterial density on the antibacterial activity of the QACs, *E. faecalis* suspensions from an overnight culture were prepared in the range from 10^6^ to 10^10^ CFU/mL in Brain Heart Infusion broth (BHI, VWR International BVBA, Leuven, Belgium) by either diluting or concentrating bacterial suspensions. The MBCs corresponding to *E. faecalis* suspensions with different initial bacterial densities were determined only for the compound A_14_ in the concentration range between 500 and 0.49 μM prepared by 2-fold serial dilutions. The MBCs were evaluated as described above after the bacteria were exposed to the QACs for 3 min, 60 min or 24 h. The growth rate of tested *E. faecalis* suspensions at different initial bacterial densities was monitored every 30 min during 7 h of incubation by measuring the optical density (*OD*_600_). The values were fitted with the logistic equation

$$N\,\left(t\right)=\frac{K\,N_{0}}{\left(K-N_{0}\right)\,e^{-r\,t}+N_{0}}$$

where *N*_0_ is the initial *OD*_600_, *K* is the carrying capacity (maximal *OD*_600_ in the stationary growth phase), *t* is the length of time of incubation, and *r* is the growth rate constant.

### Biofilm Susceptibility Assay

To grow the biofilms, an overnight culture of *E. faecalis* (average viable bacterial concentration of 1 × 10^8^ CFU/ml) was diluted 100-fold in BHI. *E. faecalis* biofilms were formed on titanium radial disks (commercially pure titanium, grade 2) with 7 mm diameter and 1 mm thickness. Prior to the experiments all disks were sandblasted (FerroEcoBlast Europe; Microblast ceramic beads B120) to expose fresh titanium surface, cleaned in 70% ethanol and autoclaved at 134°C for 20 min. The biofilms were grown for 3 days at 37°C ± 1°C without shaking or changing of media to achieve a surface coverage of approximately 25% and a surface bacterial density of ∼1.5 × 10^11^ bacteria per mL. Biofilms grown on a titanium surface were exposed to 1.5 ml of **A_14_**, **B_14_**, and **C_14_** dissolved in BHI for 1, 3, and 60 min. After treatment the individual disks were rinsed with saline solution to remove QACs as well as unattached bacteria. In laser treated biofilms samples were treated with QACs for 1 min followed by 10 s of Er:YAG – SSP laser treatment. The LightWalker Er:YAG (Fotona, Ljubljana, Slovenia) was set with the following parameters: laser wavelength 2940 nm; contact handpiece H14, Flat Sweeps fiber tip 400/14 positioned 5 mm above the biofilm disk, energy 20 mJ, frequency 15 Hz, water off, air off, single pulse modality – SSP (super short pulse – 50 μs). The titanium disks with their biofilm were positioned at the bottom of cylindrical irrigation system (7.5 mm diameter and 2 cm high) in the presence of 1.5 ml of either saline or the QAC dissolved in BHI. The antibacterial concentration was 250 μM which was the highest soluble QAC concentration in BHI broth. The rinsed biofilms were stained with 5 μL of premixed saline-diluted Syto 9 and propidium iodide stock solutions (1: 300) from LIVE/DEAD^TM^ BacLight^TM^ Bacterial Viability Kit L7012 (Thermo Fisher Scientific, Eugene, OR, United States). After 5 min of incubation in the dark the samples were observed by fluorescence microscope Zeiss Axio Observer Z1 equipped with confocal unit LSM 800 (CLSM). Z-stacks and tiles were taken at 100× or 20× magnification at three randomly selected view fields. Image acquisition and control of the microscope were performed with ZEN 2.3 (ZEISS GmbH, Germany). For the 100× magnification, blue 488 nm and yellow 561 nm lasers were set to 1% intensity, GaAsP PMT detectors to 700 V, pinhole to 71 μm, and the size of the acquired 8-bit images was 3476 × 3476 pixels. For the 20× magnification, blue 488 nm and yellow 561 nm lasers were set to 0.85% intensity, GaAsP PMT detectors to 700 V, pinhole to 50 μm and the size of acquired 8-bit images was 1306 × 1306 pixels. The typical Z-step of the Z-stack was 1 μm for 100× magnification and 3.26 μm for 20× magnification. The images were composed of four different view fields. A custom script was written (Image J) and used to evaluate the effect of treatment on the fraction of dead cells and biofilm surface coverage.

The Bliss independence model was used for the evaluation of interactions of the combined QACs and laser treatment (Bliss, 1939). The model assumes that a single agent acts independently but contributes to the final outcome (Bliss, 1939; Foucquier and Guedj, 2015). The observed combined effect (E_obs_) is compared to the expected combined effect (E_exp_) which is calculated as E_exp_ = 1 – (1 – E_1_) × (1 – E_2_), where E_1_ and E_2_ are the effects of the single treatments. The difference between the E_obs_ and E_exp_ is the Excess over Bliss (*eob*). Positive *eob* values indicate synergistic interaction, whereas negative *eob* values indicate antagonistic behavior. Null *eob* value implies no interaction.

### Cell Viability Evaluation

Standard MTT assay (3-(4,5-dimethylthiazol-2-yl)-2,5-diphenyltetrazolium bromide; Sigma-Aldrich, Prague, Czechia) was used according to the manufacturer’s protocol on the CHO-K1 (Chinese hamster ovary, ECACC, Salisbury, United Kingdom) in order to compare the effect of different compounds within the series. The cells were cultured according to ECACC recommended conditions and seeded in a density of 8000 per well as described previously (Soukup et al., 2020). Briefly, the tested compounds (series A, B, C) were dissolved in DMSO (Sigma-Aldrich, Prague, Czechia) and subsequently diluted in the Nutrient Mixture F-12 Ham growth medium (Sigma-Aldrich, Prague, Czechia) supplemented with 10% Fetal Bovine Serum and 1% Penicillin-Streptomycin (both Sigma-Aldrich, Prague, Czechia) so that the final concentration of DMSO did not exceed 0.5% (v/v). In the case of sodium hypochloride, the commercially available detergent called Savo containing this active compound was diluted to the initial half concentration (i.e., 2.35%) with the supplemented growth medium mentioned above. Thereafter, CHO-K1 cells were exposed to two-fold diluted series A, B, C or ten-fold diluted sodium hypochlorite for 24 h. Then the medium was replaced by a medium containing 0.5 mg/ml of MTT and the cells were allowed to produce formazan for approximately 3 h under surveillance. Thereafter, the medium with MTT was removed and crystals of formazan were dissolved in DMSO (100 μl/well). Cell viability was assessed spectrophotometrically by the amount of formazan produced. The absorbance was measured at 570 nm on Synergy HT (BioTek, Winooski, VT, United States). IC_50_ (half maximal inhibitory concentration) was then calculated from the control – subtracted triplicates using non-linear regression (four parameters) by GraphPad Prism 5.03 or 7.03 software (GraphPad Software Inc., San Diego, CA, United States). Final IC_50_ and SEM (standard error of the mean) values were obtained as a mean of three independent measurements.

### Statistical Analysis

All biological experiments were independently performed at least three times with three replicates for each sample. The results for the biofilm susceptibility were analyzed by Shapiro–Wilk normality test. Tukey’s multiple comparison test was used to evaluate the live/dead ratio (we have assumed that the data are normally distributed), whereas Dunn’s multiple comparison test was used for surface coverage, where the results were not normally distributed. *P*-values of <0.05 were considered to be significant. GraphPad Prism 7.03 software (GraphPad Software Inc., San Diego, CA, United States) was used for statistical analysis and graphical representation.

## Results and Discussion

### Chemistry

**FIGURE 1:**
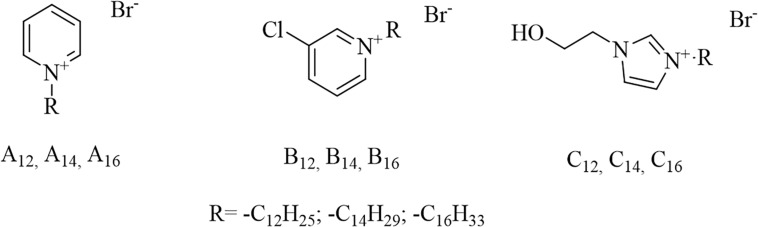
Chemical structures of antibacterial compounds used in this study. The number subscripts stand for the length of the alkyl chain (e.g., **A_12_** is 1-dodecylpyridinium, with *R* = -C_12_H_25_).

The chemical structures of 1-Alkylpyridinium derivatives (**A_12_, A_14_, A_16_**); 1-alkyl-3-chloropyridinium derivatives (**B_12_, B_14_, B_16_**), and 3-alkyl-1-(2-hydroxyethyl)imidazolium derivatives (**C_12_, C_14_, C_16_**) are shown in Figure 1.

We have prepared 9 quaternary ammonium salts containing pyridine or imidazole heterocyclic rings. The compounds of group A and C were synthesized as described previously and served as reference QACs (Marek et al., 2010; Soukup et al., 2020). The novel antibacterial compounds (B group) were prepared by Menshutkin reaction, where the tertiary amine was converted into a quaternary ammonium salt by reaction with an alkyl halide (nucleophilic substitution-type reaction). Each homologous group consisted of three *N*-alkyl derivatives with either 12, 14 or 16 carbon atoms in the alkyl chain. The structures of the new compounds were confirmed by ^1^H and ^13^C NMR and HRMS analysis. The purity of all the compounds was ≥95%. The yields, melting points, purities and Clog *P* (calculated logarithm of the partition coefficient) of different antibacterial compounds are given in Table 2. Clog *P* was calculated with MarvinSketch (version 14.9.8.0) software. As expected, the melting points of the new compounds and Clog *P* values increased with increasing alkyl chain length.

**TABLE 2:** Yields, melting points, purity and Clog *P* of antibacterial QACs used in this study.

**Compound**	**Alkyl chain**	**Yield (%)**	**Purity (%)**	**Melting point (°C)**	**Clog *P***
**A_12_**	12	82.13	95	73.0–75.0	1.693
**A_14_**	14	75.61	96	58.0–60.0	2.582
**A_16_**	16	98.76	96	63.0–64.0	3.471
**B_12_**	12	82.84	95	68.5–69.2	2.297
**B_14_**	14	69.27	99	84.0–84.6	3.186
**B_16_**	16	76.53	100	91.4–92.2	4.075
**C_12_**	12	77.76	95	Oil	0.720
**C_14_**	14	83.69	95	44.0–46.0	1.610
**C_16_**	16	17.20	95	49.2–50.1	2.499

### *In vitro* Antibacterial Activity

#### Susceptibility of QACs Against Planktonic Bacteria

**FIGURE 2:**
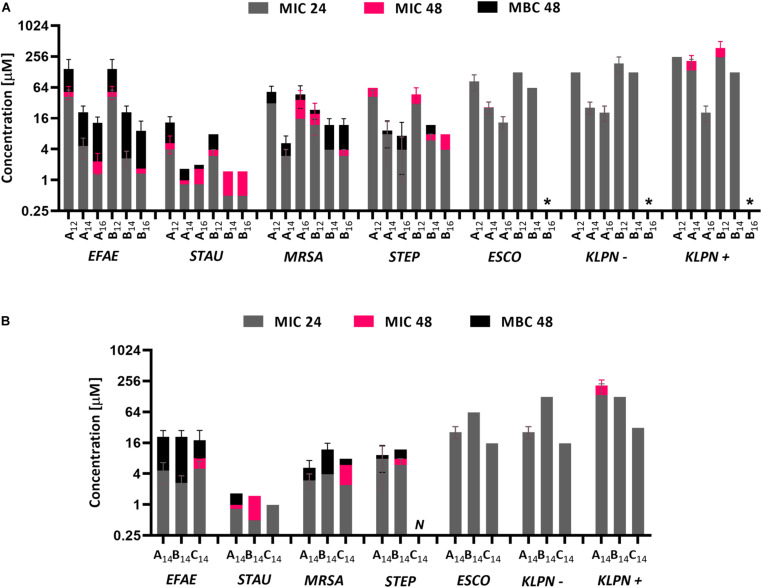
MIC and MBC values for different bacterial strains and different QACs. MIC values were determined after 24 and 48 h of incubation, whereas MBC was determined only after 48 h of incubation **(A)**. The comparison between different QACs with the same length of alkyl chain **(B)**. The results for **C_12–16_** have been already published (Soukup et al., 2020), *N* was not measured, * MIC or MBC was higher than the highest soluble concentration of the compound. Abbreviations for bacterial strains: EFAE, *E. faecalis*; STAU, *S. aureus*; MRSA, methicillin-resistant *S. aureus*; STEP, *S. epidermidis*; ESCO, *E. coli*; KLPN–, *K. pneumoniae*, KLPN+, extended-spectrum β-lactamase-producing *K. pneumoniae*.

The series of synthesized QACs were evaluated for their antibacterial activity against selected nosocomial planktonic bacteria using broth microdilution assay. The antibacterial effect of the new chlorine-substituted *N*-Alkylpyridiniums (**B_12__–__16_**) and reference *N*-Alkylpyridiniums (**A_12__–__16_**) on planktonic bacterial strains is shown in Figure 2. All the compounds were effective against the tested bacterial strains. Higher antibacterial activity, as indicated by lower MIC value, was found against the G+ bacteria *E. faecalis* (EFAE), *S. aureus* (STAU), methicillin-resistant *S. aureus* (MRSA), and *S. epidermidis* (STEP). The lowest MIC and MBC values were obtained for *S. aureus*. The observed higher susceptibility of the G+ bacteria to QACs compared to G− bacteria is in agreement with the literature (Tischer et al., 2012; Shtyrlin et al., 2016). There is a general trend of decreasing MIC values with increasing alkyl chain length for all antibacterial compounds tested. The exception is A_14_ which was more efficient against MRSA than **A_16_**. Such increasing antimicrobial activity with increasing alkyl chain length has been reported previously (Li et al., 2013; Zhang et al., 2016). When an *N*-Alkylpyridinium QAC was compared with a chlorine-substituted *N*-alkylpyridinium or *N*-Alkylimidazolium at a given alkyl chain length (i.e., **A_14_**, **B_14_**, and **C_14_** in Figure 2), there was no significant difference in the antimicrobial activity for G+ bacteria. In the case of the G− bacteria *E. coli* (ESCO), *K. pneumoniae* (KLPN−), and extended-spectrum β-lactamase-producing *K. pneumoniae* (KLPN+), the most effective were imidazolium compounds. Our results are consistent with previous observations that small changes in the structure of QACs such as introduction of electronegative atom allow for fine-tunability of surfactant properties (Brown et al., 2017; Fuente-Nunez et al., 2018).

#### The Effect of the Initial Bacterial Density on Planktonic Antimicrobial Effectiveness

**FIGURE 3:**
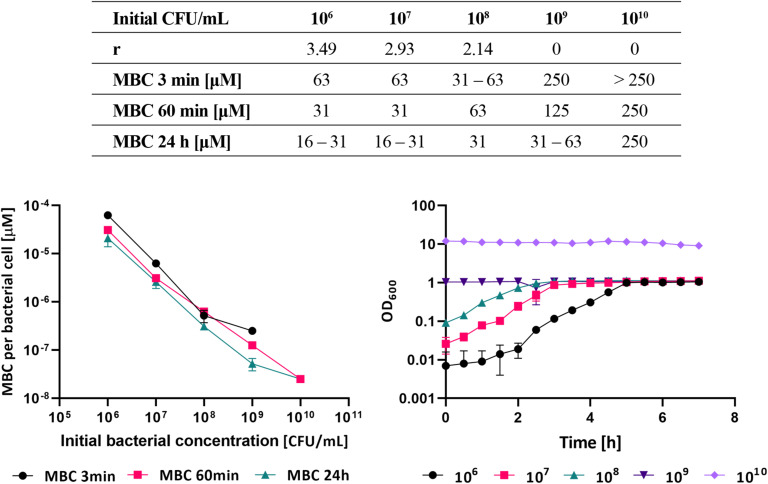
The growth rates *r* and experimentally determined MBCs at different initial *E. faecalis* culture densities. MBCs were determined after 3 min, 60 min, and 24 h of exposure to **A_14_ (upper table)**. The relative MBC values of **A_14_** antibacterial compound calculated per single bacterial cell **(left bottom graph)**. Growth curves of *E. faecalis* suspensions at different initial bacterial densities in the absence of **A_14_ (right bottom graph)**.

The effect of different initial bacterial densities on the MBC are given in Figure 3. The MBC was determined after 3 min, 60 min, and 24 h of bacterial exposure to the antibacterial compound. The MBC increased with the initial bacterial density. This is usually interpreted as an increased resistance to the antibiotic due to the induction of quorum sensing pathways [i.e., by increasing the number of persistent cells in the population, the expression of peroxidases which provide protection against reactive oxygen species, or the overexpression of an efflux pump (Rémy et al., 2018)]. The increase in MBC with cell density, however, was much less than the increase in the number of bacterial cells. If one calculates the relative MBC value per bacterial cell the effective MBC in fact significantly decreases with cell densities (Figure 3). The required MBC after 24 h at 10^6^ CFU/ml initial bacterial density was 2.1 × 10^–5^ μM and decreased to 2.7 × 10^–8^ μM at the initial bacterial density of 10^10^ CFU/ml. This is more than three orders of magnitude. The results imply that the denser the initial bacterial suspension the more the individual bacterial cell is susceptible to the antibacterial compound. This is surprising and could be due to an increased level of stress experienced by the individual cell in a more crowded environment. Although at high cell densities bacteria may collectively adapt their behavior and increase antibiotic resistance the results of this study suggest that the individual cells of *E. faecalis* become more stressed and susceptible to the antibiotic at higher initial bacterial concentrations. This could be due to reduced availability of nutrients and reduced growth rate. To check this we have grown bacteria at different initial densities in fresh medium. As illustrated in Figure 3, the optical density increased during incubation for the dilute initial bacterial suspensions (i.e., from 10^6^ to 10^8^ CFU/ml), but remained unchanged if the initial bacterial concentration was 10^9^ CFU/ml or higher. The growth rate, which is a general indicator of cell well-being, decreased significantly with increasing initial cell density.

#### Effectiveness of QACs Against Biofilm

**FIGURE 4:**
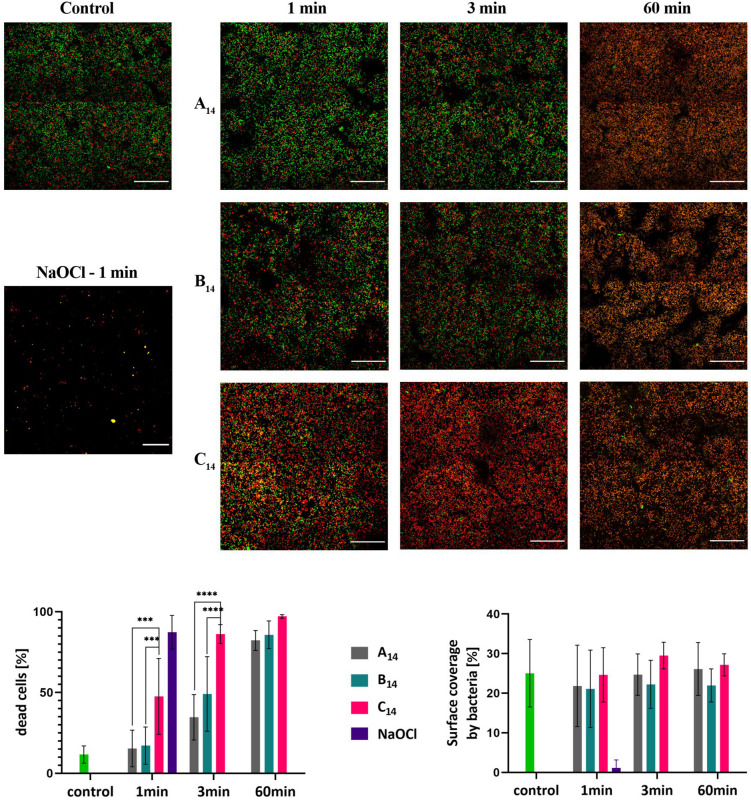
**Up:** CLSM images of *E. faecalis* biofilms grown on a titanium surface for 72 h at 37°C. In the left column are the live/dead stained biofilms for a control biofilm treated with 0.9% NaCl solution, and a positive control treated with 3% NaOCl for 1 min. The biofilms on the right are after 1, 3, and 60 min of treatment with **A_14_**, **B_14_**, and **C_14_** at a concentration of 250 μM. Live bacteria are green, dead are red fluorescent. For each antibacterial and length of treatment, the representative view fields are shown in the panel. The scale bar on the micrographs represents 50 μm. **Down:** The effect of QACs on the fraction of dead cells in the biofilm **(left bottom graph)**, and surface covered by bacteria **(right bottom graph)**. The average values and standard deviations are given (*n* = 9–27). Significance *** (*p* < 0.001); **** (*p* < 0.0001).

The effectiveness of different QACs against *E. faecalis* biofilms was determined with **A_14_**, **B_14_**, and **C_14_** compounds as shown in Figure 4. All tested QACs were effective against *E. faecalis* biofilms after 60 min of treatment. The fraction of dead (red) cells increased with the time of incubation. The increase was most pronounced for **C_14_**, where the majority of cells in the biofilm were dead already after 1 min of treatment. The effectiveness of **B_14_** was higher than that of **A_14_**. As a positive control we have used Sodium hypochlorite solution applied at the concentration commonly used in clinical practice (3% V/V). After 1 min of exposure the biofilm coverage decreased below 1%. The applied NaOCl concentration was approximately 150 times higher than QACs’ concentrations used in this study.

To quantify the effect of different QACs on *E. faecalis* biofilms we have determined the fraction of dead cells and the biofilm surface coverage prior to and after the treatment (Figure 4). Prior to treatment, most of the bacterial cells in a biofilm (88 ± 6%) were alive. After 60 min of treatment, the proportions of live cells decreased dramatically and were 18, 14, and 3% for **A_14_**, **B_14_**, and **C_14_**, respectively. The most effective compound against biofilm bacteria was **C_14_**, where after 3 min of treatment the fraction of dead bacteria was already 86 ± 4%. **B_14_** was more effective than **A_14_**. It is interesting to note that surface coverage did not change significantly during the treatment, suggesting that **A_14_**, **B_14_**, and **C_14_** kill bacterial cells in a biofilm, but do not remove them from the surface. Similar behavior has been observed for chlorhexidine treatment, where the bacteria in the biofilm were killed, but not removed from the dentin surface (Paz et al., 2010).

#### Comparison of the Effectiveness of QACs Against Planktonic and Biofilm Cells

In plankton suspensions we were able to reach bactericidal concentration for all tested QACs. The situation was quite different in biofilms. The concentrations that work in plankton were not sufficient to kill all bacteria in the biofilm. It is usually assumed that antibacterials are significantly less effective in biofilms compared to plankton and up to 1000-fold higher concentrations were reported for biofilms compared to plankton (Ceri et al., 1999; Olson et al., 2002; ASTM International, 2017). Unfortunately, due to the solubility limit, it was not possible to increase the concentration of the QACs to concentrations that would kill all bacteria in the biofilm. Nevertheless, we can compare the effectiveness in plankton and biofilm if we decrease the concentration of biocide in the plankton to match the fraction of the dead cells in the biofilm. The less we have to dilute the concentration of biocide in plankton to match the fraction of dead bacteria in biofilm the more potent the compound is as a biocide in the biofilm. The experiment was performed at high density *E. faecalis* suspensions (2.4 × 10^9^ CFU/mL) to mimic the high density of bacteria in a biofilm. By comparing QACs effectiveness at high initial planktonic densities we have largely eliminated the effect of the bacterial density and estimated the effect of biofilm induced changes on QACs sensitivity. One has to be careful, however, as bacterial cells in plankton differ from biofilm not only in cell density but also in cell metabolism and extracellular matrix viscoelasticity. To match the fraction of dead cells in the biofilm, the 10-fold dilution of compounds **A_14_** and **B_14_** were needed, whereas only 4-fold dilution of compound **C_14_** was required. This indicates that **C_14_** has a better activity in the biofilm compared to **A_14_** and **B_14_**. The activity of QACs in biofilms relative to plankton was two orders of magnitude lower than usually reported in the literature. Although our biofilms were mature, they were relatively thin (up to 10 μm) which may explain the relatively high efficiency of the QACs against biofilms. Similar observations have been made for *S. mutans* biofilms, where biofilm cells were only 8 times less susceptible to QACs compared to plankton bacteria (López Pérez et al., 2017).

#### Er:YAG Photoacoustic Irrigation With QACs in Biofilm Treatment

**FIGURE 5:**
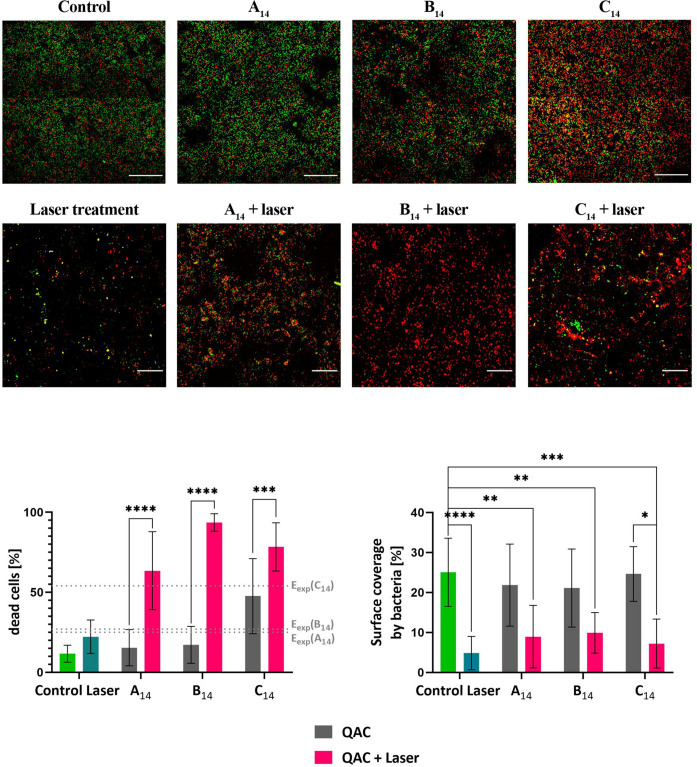
**Up:** CLSM images of *E. faecalis* biofilms grown on a titanium surface for 72 h at 37°C. **Upper row:** live/dead stained biofilms of chemically treated biofilms after 1 min of exposure to **A_14_**, **B_14_**, and **C_14_**; **lower row:** biofilms exposed to **A_14_**, **B_14_**, and **C_14_** for 1 min followed by 10 s of Er:YAG photoacoustic irrigation. Control: treatment with 0.9% NaCl solution. Laser treatment Er:YAG treatment of biofilms in 0.9% NaCl solution. Live bacteria are green, dead are red fluorescent. The representative field views are shown. The scale bar on micrographs represents 50 μm. **Down left:** The effect of each type of treatment on the fraction of dead cells. The expected combined effect (E_exp_) of QACs and laser treatments according to the Bliss Independence model is indicated by doted lines. **Down right:** Surface covered with bacteria after different treatments. The average values and standard deviations are given (*n* = 9–27). Significance * (*p* < 0.05); ** (*p* < 0.01); *** (*p* < 0.001); **** (*p* < 0.0001).

Although treatment with QACs killed most of the bacteria in biofilms, a significant fraction survived (e.g., 18% with the **A_14_** compound after 60 min of treatment). Since 60 min is far too long for many applications (e.g., in dentistry), we tested if laser treatment could potentiate the effect of QACs. To check this we pretreated the biofilms chemically with the QAC for 1 min followed by Er:YAG photoacoustic streaming treatment for 10 s (Figure 5). As a control, biofilms were pretreated with 0.9% NaCl saline solution followed by laser treatment. In the control samples the Er:YAG photoacoustic streaming substantially decreased the biofilm surface coverage, but did not change the fraction of dead bacteria in the biofilms. It is important to note that in photoacoustic streaming with short laser pulses the laser is not acting directly on the surface but it induces cavitation and consequently increases streaming in the fluid, which removes bacteria from the surface but does not change the ratio of live/dead bacteria. On the other hand, when samples were pretreated with QACs followed by laser treatment the fraction of dead cells increased noticeably.

To quantify the data, the proportion of dead cells and the surface coverage after Er:YAG photoacoustic irrigation were determined and are shown in Figure 5. If the cells were in saline solution the laser treatment significantly decreased the surface coverage, but did not significantly change the ratio of live/dead bacteria which remained on the surface. Although the surface coverage decreased more in saline solution compared to QACs the difference was not significant. On the other hands, when biofilms were chemically pretreated with QACs the fraction of dead cells increased significantly after laser treatment, for all tested QACs. To check if the potentiation provided by laser treatment is synergistic or additive, we have determined the Bliss independence index. In all cases it was positive indicating a synergistic action of QACs and laser treatment. The synergistic effect was most pronounced with the novel **B_14_**. Taken together, these data imply that short laser treatment dramatically improves the effectiveness of QACs by removing bacteria from the surface and by increasing the killing rate. This may allow a shorter chemical exposure time and lower dosage of QACs used in applications.

### Cytotoxicity of QACs

*In vitro* cytotoxicity evaluation of QACs on mammalian CHO-K1 (Chinese hamster ovary) cell line is given in Table 3. With the increase in the alkyl chain length the cytotoxic potential of the drugs increased. This effect is probably caused due to the increasing lipophilicity expressed as the Clog *P* (Table 2) and correlates most likely with the ability to penetrate into cells more easily (Marek et al., 2015; Soukup et al., 2020).

**TABLE 3:** The effect of the tested compounds on the CHO-K1 cell viability.

**Compound**	**IC_50_ ± SEM (μ M)**
A_12_^a^	38.80 ± 0.9
A_14_^a^	24.70 ± 0.2
A_16_^a^	16.70 ± 0.2
B_12_	25.50 ± 2.5
B_14_	17.17 ± 1.3
B_16_	12.54 ± 1.1
C_12_^b^	1.43 ± 3.0
C_14_^b^	1.19 ± 0.9
C_16_^b^	1.03 ± 1.0
Sodium hypochlorite	6.45 ± 1.1

*Values are expressed as the IC_50_: Mean ± SEM (*n* = 3). ^a^The preparation of **A_12–16_** has been described elsewhere (Marek et al., 2010). ^b^The preparation and the biological assessment of **C_12–16_** have been described elsewhere (Soukup et al., 2020).*

The highest cytotoxicity was determined in the series C which is most likely due to the hydroxyethyl group in the structure. The chlorine substituent in the meta position within the series B caused higher cytotoxicity in comparison to series A. The sodium hypochlorite was more cytotoxic than A and B compounds. The cytotoxicity of commonly used or structure modified QACs was already studied several times. However, the comparison of the obtained values with already published is limited due to the assays’ differences, especially regarding to the type of the cells used in experiments. Nevertheless, the cytotoxicity of the well-known QACs representative, benzalkonium chloride, is frequently established in similar range as our QACs [EC_50_ ∼ 12.42 μM for human hepatoma cell line (Christen et al., 2017); IC_50_ ∼ 14.8 μM for Human alveolar cells (Kwon et al., 2019); IC_50_ ∼ 22.8 μM for osteosarcoma cybrid cells (Datta et al., 2017)]. On the other hand, lower toxicity was previously described for QAC-like magnetic and non-magnetic ionic liquid surfactants and their polymeric analogs (∼50 μM for human embryonic kidney cells) (Fuente-Nunez et al., 2018).

## Conclusion

All the selected nosocomial bacterial species were susceptible to the tested QACs in the planktonic state. The novel chlorine substituted *N*-alkylpyridinium (**B_12__–__16_**) compounds were most effective against planktonic *S. aureus* with MIC values between 0.5 and 4 μM. The new QACs have an effect comparable to the effect of the non-chlorinated derivatives of *N*-alkylpyridinium and *N*-Alkylimidazolium. The activity of QACs on planktonic bacteria was dependent on the initial bacterial concentration in the suspension. By increasing the initial bacterial concentration, which leads to increased bacterial density during the growth, the level of stress experienced by individual cell was increased. This resulted in lowering the effective QAC concentration needed to kill a bacterial cell. All evaluated QACs demonstrate anti-biofilm activity against *E. faecalis* biofilms grown on a titanium surface. The best anti-biofilm compound was 3-tetradecyl-1-(2-hydroxyethyl)imidazolium bromide (**C_14_**) which was the most cytotoxic. Compared to planktonic bacteria, the biofilm bacteria were only 4-fold more resistant to **C_14_**. The most significant finding of this study is that a short 10 s treatment of Er:YAG – SSP photoacoustic steaming irrigation in the presence of new *N*-alkylpyridinium improved significantly its anti-biofilm action. In particular, it decreased the surface coverage of the biofilm and significantly increased the fraction of dead bacteria. The application of QACs supported by photoacoustic irrigation could be a promising new strategy in combating biofilm-related problems.

## Data Availability Statement

The raw data supporting the conclusions of this article will be made available by the authors, without undue reservation.

## Ethics Statement

This study uses strains that were isolated from the samples obtained in the clinical laboratory at the University Hospital Hradec Kralove. The University Hospital Hradec Kralove did not require the study to be reviewed or approved by an Ethics Committee because the clinical strains used in this study come from the routine procedures in the clinical laboratory.

## Author Contributions

MH performed biological experiments, result analysis, conducted the work and was involved in the designing of the work, writing and interpretation of the data. ST was participating on biological experiments, designing of the work, writing and interpretation of the data. AM performed synthesis and participated in writing. LPr performed NMR and HPLC analysis. LPu performed cytotoxicity experiments. MB participate in writing and interpretation of the data. ID was involved in CSLM imagining, data analysis and writing. JM participated in synthesis, writing and interpretation of the data. DS was involved in designing of the work, writing, interpretation of the data and submission of the manuscript.

## Conflict of Interest

The authors declare that the research was conducted in the absence of any commercial or financial relationships that could be construed as a potential conflict of interest.

## References

[B1] Akcay M. Arslan H. Mese M. Durmus N. Capar I. D. (2017). Effect of photon-initiated photoacoustic streaming, passive ultrasonic, and sonic irrigation techniques on dentinal tubule penetration of irrigation solution: a confocal microscopic study. *Clin. Oral Investig.* 21 2205–2212. 10.1007/s00784-016-2013-y 27921170

[B2] ASTM International (2017). *ASTM E2799-17, Standard Test Method for Testing Disinfectant Efficacy Against Pseudomonas aeruginosa Biofilm using the MBEC Assay.* West Conshohocken, PA: ASTM International.

[B3] Bliss C. I. (1939). The toxicity of poisons applied jointly. *Ann. Appl. Biol.* 26 585–615. 10.1111/j.1744-7348.1939.tb06990.x

[B4] Boutsioukis C. Verhaagen B. Versluis M. Kastrinakis E. Wesselink P. R. van der Sluis L. W. M. (2010). Evaluation of irrigant flow in the root canal using different needle types by an unsteady computational fluid dynamics model. *J. Endod.* 36 875–879. 10.1016/j.joen.2009.12.026 20416437

[B5] Brown P. Sresht V. Eral B. H. Fiore A. Fuente-Núñez C. O’Mahony M. (2017). CO2 -reactive ionic liquid surfactants for the control of colloidal morphology. *Langmuir* 33 7633–7641. 10.1021/acs.langmuir.7b00679 28699755

[B6] Ceri H. Olson M. E. Stremick C. Read R. R. Morck D. Buret A. (1999). The calgary biofilm device: new technology for rapid determination of antibiotic susceptibilities of bacterial biofilms. *J. Clin. Microbiol.* 37 1771–1776. 10.1128/jcm.37.6.1771-1776.1999 10325322 PMC84946

[B7] Christen V. Faltermann S. Brun N. R. Kunz P. Y. Fent K. (2017). Cytotoxicity and molecular effects of biocidal disinfectants (quaternary ammonia, glutaraldehyde, poly(hexamethylene biguanide) hydrochloride PHMB) and their mixtures in vitro and in zebrafish eleuthero-embryos. *Sci. Total Environ.* 586 1204–1218. 10.1016/j.scitotenv.2017.02.114 28236482

[B8] Clarkson R. M. Moule A. J. (1998). Sodium hypochlorite and its use as an endodontic irrigant. *Aust. Dent. J.* 43 250–256. 10.1111/j.1834-7819.1998.tb00173.x 9775472

[B9] CLSI (2018). *Methods for Dilution Antimicrobial Susceptibility Tests for Bacteria that Grow Aerobically: CLSI Standard M07*, 11th Edn Wayne, PA: Clinical and Laboratory Standards Institute.

[B10] Courvalin P. (2016). Why is antibiotic resistance a deadly emerging disease? *Clin. Microbiol. Infect.* 22 405–407. 10.1016/j.cmi.2016.01.012 26806259

[B11] Datta S. Baudouin C. Brignole-Baudouin F. Denoyer A. Cortopassi G. A. (2017). The eye drop preservative benzalkonium chloride potently induces mitochondrial dysfunction and preferentially affects LHON mutant cells. *Investig. Opthalmology Vis. Sci.* 58:2406. 10.1167/iovs.16-20903 28444329 PMC5407244

[B12] Dolezal R. Soukup O. Malinak D. Savedra R. M. L. Marek J. Dolezalova M. (2016). Towards understanding the mechanism of action of antibacterial N-alkyl-3-hydroxypyridinium salts: biological activities, molecular modeling and QSAR studies. *Eur. J. Med. Chem.* 121 699–711. 10.1016/j.ejmech.2016.05.058 27341309

[B13] Foucquier J. Guedj M. (2015). Analysis of drug combinations: current methodological landscape. *Pharmacol. Res. Perspect.* 3:e00149. 10.1002/prp2.149 26171228 PMC4492765

[B14] Fuente-Nunez C. Brown P. Torres M. D. T. Cao J. Lu T. K. (2018). Magnetic surfactant ionic liquids and polymers with tetrahaloferrate (III) anions as antimicrobial agents with low cytotoxicity. *Colloid Interface Sci. Commun.* 22 11–13. 10.1016/j.colcom.2017.11.002

[B15] Gerba C. P. (2015). Quaternary ammonium biocides: efficacy in application. *Appl. Environ. Microbiol.* 81 464–469. 10.1128/AEM.02633-14 25362069 PMC4277564

[B16] Jennings M. C. Ator L. E. Paniak T. J. Minbiole K. P. C. Wuest W. M. (2014). Biofilm-eradicating properties of quaternary ammonium amphiphiles: simple mimics of antimicrobial peptides. *ChemBioChem* 15 2211–2215. 10.1002/cbic.201402254 25147134

[B17] Khan H. A. Baig F. K. Mehboob R. (2017). Nosocomial infections: epidemiology, prevention, control and surveillance. *Asian Pac. J. Trop. Biomed.* 7 478–482. 10.1016/j.apjtb.2017.01.019

[B18] Kurzmann C. Meire M. A. Lettner S. Farmakis E. T. R. Moritz A. De Moor R. J. G. (2019). The efficacy of ultrasonic and PIPS (photon-induced acoustic streaming) irrigation to remove artificially placed dentine debris plugs out of an artificial and natural root model. *Lasers Med. Sci.* 35 719–728. 10.1007/s10103-019-02912-3 31782022

[B19] Kwon D. Lim Y. Kwon J. Shim I. Kim E. Lee D. (2019). Evaluation of pulmonary toxicity of benzalkonium chloride and triethylene glycol mixtures using in vitro and in vivo systems. *Environ. Toxicol.* 34 561–572. 10.1002/tox.22722 30786124 PMC6594094

[B20] Li F. Weir M. D. Xu H. H. K. (2013). Effects of quaternary ammonium chain length on antibacterial bonding agents. *J. Dent. Res*. 92 932–938. 10.1177/0022034513502053 23958761 PMC3775374

[B21] López Pérez D. Baker P. J. Pintar A. L. Sun J. Lin N. J. Lin-Gibson S. (2017). Experimental and statistical methods to evaluate antibacterial activity of a quaternary pyridinium salt on planktonic, biofilm-forming, and biofilm states. *Biofouling* 33 222–234. 10.1080/08927014.2017.1286476 28270052

[B22] Lukač N. Jezeršek M. (2018). Amplification of pressure waves in laser-assisted endodontics with synchronized delivery of Er:YAG laser pulses. *Lasers Med. Sci.* 33 823–833. 10.1007/s10103-017-2435-z 29327088 PMC5911281

[B23] Marek J. Malinak D. Dolezal R. Soukup O. Pasdiorova M. Dolezal M. (2015). Synthesis and disinfection effect of the pyridine-4-aldoxime based salts. *Molecules* 20 3681–3696. 10.3390/molecules20033681 25719739 PMC6272478

[B24] Marek J. Stodulka P. Cabal J. Soukup O. Pohanka M. Korabecny J. (2010). Preparation of the pyridinium salts differing in the length of the N-Alkyl substituent. *Molecules* 15 1967–1972. 10.3390/molecules15031967 20336025 PMC6257284

[B25] Olivi G. DiVito E. Peters O. Kaitsas V. Angiero F. Signore A. (2014). Disinfection efficacy of photon-induced photoacoustic streaming on root canals infected with *Enterococcus faecalis*. *J. Am. Dent. Assoc.* 145 843–848. 10.14219/jada.2014.46 25082933

[B26] Olson M. E. Ceri H. Morck D. W. Buret A. G. Read R. R. (2002). Biofilm bacteria: formation and comparative susceptibility to antibiotics. *Can. J. Vet. Res.* 66 86–92. 11989739 PMC226988

[B27] Paz L. E. C. Bergenholtz G. Svensäter G. (2010). The effects of antimicrobials on endodontic biofilm bacteria. *J. Endod.* 36 70–77. 10.1016/j.joen.2009.09.017 20003938

[B28] Rémy B. Mion S. Plener L. Elias M. Chabrière E. Daudé D. (2018). Interference in bacterial quorum sensing: a biopharmaceutical perspective. *Front. Pharmacol.* 9:203. 10.3389/fphar.2018.00203 29563876 PMC5845960

[B29] Shtyrlin N. V. Sapozhnikov S. V. Galiullina A. S. Kayumov A. R. Bondar O. V. Mirchink E. P. (2016). Synthesis and antibacterial activity of quaternary ammonium 4-deoxypyridoxine derivatives. *BioMed Res. Int.* 2016:3864193. 10.1155/2016/3864193 27800491 PMC5069379

[B30] Soukup O. Benkova M. Dolezal R. Sleha R. Malinak D. Salajkova S. (2020). The wide-spectrum antimicrobial effect of novel N-alkyl monoquaternary ammonium salts and their mixtures; the QSAR study against bacteria. *Eur. J. Med. Chem*. 206:112584. 10.1016/j.ejmech.2020.112584 32853858

[B31] Tischer M. Pradel G. Ohlsen K. Holzgrabe U. (2012). Quaternary ammonium salts and their antimicrobial potential: targets or nonspecific interactions? *ChemMedChem* 7 22–31. 10.1002/cmdc.201100404 22113995

[B32] Watkins K. (2018). Emerging infectious diseases: a review. *Curr. Emerg. Hosp. Med. Rep.* 6 86–93. 10.1007/s40138-018-0162-9 32226656 PMC7100414

[B33] Zehnder M. (2006). Root canal irrigants. *J. Endod.* 32 389–398. 10.1016/j.joen.2005.09.014 16631834

[B34] Zhang K. Cheng L. Weir M. D. Bai Y.-X. Xu H. H. (2016). Effects of quaternary ammonium chain length on the antibacterial and remineralizing effects of a calcium phosphate nanocomposite. *Int. J. Oral Sci.* 8 45–53. 10.1038/ijos.2015.33 27025265 PMC4822178

[B35] Zhang Y. Chen Y. Hu Y. Huang F. Xiao Y. (2018). Quaternary ammonium compounds in dental restorative materials. *Dent. Mater. J.* 37 183–191. 10.4012/dmj.2017-096 29225280

